# Assessment of Aggregation Frameworks for Composite Indicators in Measuring Flood Vulnerability to Climate Change

**DOI:** 10.1038/s41598-019-55994-y

**Published:** 2019-12-18

**Authors:** Hyun Il Choi

**Affiliations:** Department of Civil Engineering, Yeungnam University, Gyeongsan, 38541 Korea

**Keywords:** Natural hazards, Hydrology

## Abstract

The IPCC Third Assessment Report presents a conceptual framework for vulnerability to climate change with the three attribute components of exposure, sensitivity, and coping. Since the vulnerability assessments have been conducted mainly by the composite indicators aggregated from the IPCC’s components, it is necessary to assess aggregation frameworks for constructing the composite indicators that have an influence on vulnerability assessment outcomes. This study therefore investigates the robustness of assessment outcomes for flood vulnerability to climate change through a comparative analysis of the six vulnerability indicators aggregated from the IPCC’s components by the conventional aggregation frameworks. The comparative analysis has been illustrated through both the possible combinations of reference values for vulnerability attribute components and a case study on the flood vulnerability assessment to climate change for coastal areas in the Republic of Korea. The study demonstrates that there can be large fluctuations and reversals in ranking orders across the six vulnerability outcomes by different aggregation frameworks. It concludes that for flood vulnerability assessment to climate change in coastal areas, the vulnerability indicator needs to be aggregated by a multiplicative utility function from all the three assessment components with positive elasticity to vulnerability.

## Introduction

Climate change is one of the greatest environmental threats, already having visible impacts on all countries worldwide; thus, vulnerability assessment to climate change has become one of the most important tools for providing fundamental information on mitigation and adaptation measures concerning climate variability, changes, and impacts^1^. One of the most outstanding definitions and frameworks for vulnerability assessment to climate change is discussed in the Intergovernmental Panel on Climate Change Third Assessment Report (IPCC TAR)^1^, which states that the vulnerability assessment components are exposure, sensitivity (also fragility or susceptibility), and coping (also response capacity, adaptive capacity, or resilience). This has also been described in some selected literature, including the IPCC Fourth and Fifth Assessment Reports^2–5^. As climate change has a large impact on many sectors important to society and the environment, this conceptual framework for vulnerability to climate change has been used in various vulnerability assessments for the purpose of targeting multi-dimensional issues in a wide range of fields^6^. In particular, many coastal areas have already experienced severe weather conditions, such as strong winds, heavy rains, and huge storm surges, due to climate change, causing devastating flooding in low-lying areas of coastal regions^1,4,5^. Hence, it is necessary to develop flood damage mitigation strategies in coastal regions through an appropriate assessment of flood vulnerability that indicates a degree of potential damage at flood risk.

Vulnerability to climate change has been evaluated mainly with a composite indicator compiled from multiple assessment components by one of the various aggregation frameworks and methods^7^. A single composite indicator is usually compiled from individual component indicators to measure the multidimensional concept^8^, as multiple-criteria decision making or analysis is required to evaluate multidimensional issues and multiple conflicting criteria in decision making^9^. Hence, multi-attribute utility functions are most commonly and powerfully used to combine assessment attribute components into a composite indicator for the overall performance information that can provide ranked alternatives for deterministic policy and decision problems^7,8^. The general procedure for constructing a composite indicator for policy and decision making is as follows:^7^ 1) a theoretical framework is established to select and classify appropriate assessment components that fulfill the purpose of generating a meaningful composite indicator; 2) proxy variables relevant to individual components are selected and identified by multivariate analysis in the context of availability, measurability, representatives, soundness, and mutual independence; 3) a selected normalization method transforms variables measured at different units or scales into a common domain to affect the analysis in an equal scale; 4) reasonable weightings are estimated and assigned for the importance of the associated variable based on correlations between variables and the phenomena; 5) a multi-attribute utility function is employed to aggregate individual attribute components into a composite indicator to measure overall performance; and 6) sensitivity and robustness analysis is conducted to prevent the derived composite indicator from providing false or misleading information.

Since there is no prominent scheme or standard method for constructing the vulnerability composite indicator from individual attribute components, vulnerability to many aspects of natural and social sciences and humanities has been estimated somewhat discretionally by simply selecting one of several aggregation frameworks and methods. Many vulnerability assessment studies have presented various vulnerability composite indicators generated from the constituent components by either additive utility functions or multiplicative utility forms conventionally and in general^7^. Some of the previous studies addressed multiple frameworks for constructing vulnerability composite indicators^10,11^, but it is deemed acceptable in most studies that a single aggregation method is simply selected without considering the purpose and characteristics of each vulnerability assessment. Although there is limited comparison of the vulnerability outcomes from different aggregation methods by using only the correlation coefficients between them^10,11^, the robustness and sensitivity of vulnerability scores with varied types of frameworks and utility functions have not been explicitly explored.

The aim of this study is to evaluate various kinds of composite indicator products for assessing vulnerability to climate change. It uses different conventional aggregation frameworks to better understand the importance of composite indicators used in decision making for selecting alternatives or a preferred solution. Therefore, for evaluating the various composite indicators, this study investigates how frameworks of assessment components and multi-attribute utility forms for combining them affect the vulnerability composite indicator outcomes to estimate the overall performance. This study considers that all the attribute components are normalized into a commensurate scale from 1 to 100, indicating the least to most preferred, and weighted equally to individual components in order to avoid any other effects of normalization and weightings on composite indicator outcomes. It is feasible to assign equal weights to all assessment indicators in the absence of information or consensus for different weights^7^. The comparative analysis has focused on the vulnerability ranking fluctuations across the six composite indicators by the conventional aggregation frameworks to facilitate the understanding of the interaction among the constituent components and assessment outcomes with respect to various multi-attribute utility forms. The analysis is illustrated by tackling the possible reference cases for combinations of the vulnerability attribute components and by a case study based on actual observations and data in multidimensional situations for the coastal flood vulnerability assessment to climate change in the Republic of Korea.

## Conventional Frameworks of Vulnerability Indicators

When individual attribute indicators cannot satisfactorily represent the multidimensional concept, they are compiled into a composite indicator that can measure the complex phenomenon^8^. It is obvious that the aggregation frameworks and multi-attribute utility functions for individual assessment components can directly affect composite indicator outcomes, although it is also important to appropriately utilize other factors such as proxy variables, normalization methods, weighting methods, etc. Given that weighted and normalized individual attributes are under mutually independent conditions, they can be in general and conventional combined into a composite indicator by either the linear additive utility function^7^ as:

1 $$CA=\mathop{\sum }\limits_{i=1}^{n}{\alpha }_{i}{\chi }_{i}$$

or the nonlinear multiplicative utility function^7^ as:

2 $$CM=\mathop{\prod }\limits_{i=1}^{n}{\chi }_{i}^{{\beta }_{i}}$$

where the composite indicator *CA* or *CM* is additively or multiplicatively aggregated from individual weights *α*_*i*_ or *β*_*i*_, respectively, and component indicator scores *χ*_*i*_ of attribute *i* in a common scale for component numbers *n*.

After the attempted vulnerability assessment using an overall outcome from a general function that integrates the IPCC’s three components independently as representatively suggested by Adger^2^, many vulnerability indicators have been aggregated from the IPCC’s three components by the conventional utility functions. The vulnerability indicator to climate change can be expressed in Eq. (3) by an additive aggregation form as presented in select previous studies^12–14^:

3 $$V{A}_{1}={\alpha }_{1}E+{\alpha }_{2}S-{\alpha }_{3}C$$

and the vulnerability indicator to climate change can be expressed in Eq. (4) by a multiplicative aggregation form as presented in select previous studies^15–17^:

4 $$V{M}_{1}=\frac{{E}^{{\beta }_{1}}\times {S}^{{\beta }_{2}}}{{C}^{{\beta }_{3}}}\,$$

where *E*, *S*, and *C* are the three vulnerability assessment components of exposure, sensitivity, and coping, respectively. *VA*_1_ and *VM*_1_ are vulnerability composite indicators by additive and multiplicative forms, respectively. The equal weighting factors can usually be assumed for each component as used in some previous studies^12–17^. Note that in the functional relationship between attribute components and the vulnerability indicator, higher coping measures contributing to less vulnerable situations can decrease the overall vulnerability value, while higher scores of exposure and sensitivity contribute to the increasing composite indicator^3^. Thus, the coping score with a negative elasticity is employed as a subtrahend in Eq. (3) and a divisor in Eq. (4).

Given that lack of coping *LC* with a positive elasticity to vulnerability is substituted opposite to coping *C*, Eq. (3) can be rewritten by following some selected previous studies^18–20^ as:

5 $$V{A}_{2}={\alpha }_{1}E+{\alpha }_{2}S+{\alpha }_{3}LC$$

and Eq. (4) can be rewritten by following the selected previous studies^21–23^ as:

6 $$V{M}_{2}={E}^{{\beta }_{1}}\times {S}^{{\beta }_{2}}\times L{C}^{{\beta }_{3}}\,$$

Note that the lack of coping score corresponds to the reversal value of the coping attribute^22^. The equal weighting method can usually be employed as used in the selected previous studies^18–23^.

Besides the above frameworks for aggregating the IPCC’s three components individually, the vulnerability assessments have been also conducted by several modified frameworks that use various combinations of two of the IPCC’s three components. Based on the basic concept addressed in the IPCC TAR^1^, Metzger *et al*.^24^ proposed another framework that uses the potential impact component by combining the exposure and sensitivity components. For assessing the relative vulnerability of communities to climate change impacts, Hahn *et al*.^25^ developed a framework for the livelihood vulnerability index, where a function of the exposure and coping components is combined with the sensitivity component. In the MOVE (Method for the Improvement of Vulnerability in Europe) framework, the average of the sensitivity and coping components is combined with the exposure component as in Depietri *et al*.^26^ On behalf of the three modified frameworks where the two components are combined to be compiled with the other component, the framework using the potential impact *PI* as a composite component of exposure and sensitivity is selected for one of the conventional frameworks for the vulnerability indicator. Thus, Eq. (5) can be modified according to some previous studies^27–29^ as follows:

7 $$V{A}_{3}={\alpha }_{4}PI+{\alpha }_{5}LC$$

where *PI* = *α*_6_*E* + *α*_7_*S*, and Eq. (6) can be modified by following the selected previous studies^30–32^ as:

8 $$V{M}_{3}=P{I}^{{\beta }_{4}}\times L{C}^{{\beta }_{5}}$$

where *PI* = *E*^*β*6^ × *S*^*β*7^. When the equal weighting method was used in some previous studies^27,30–32^, lack of coping was more weighted than the other two components. Given the equal weights of 1/2, the vulnerability composite indicators *VA*_3_ and *VM*_3_ are aggregated from lack of coping weighted by 1/2 and exposure and sensitivity with 1/4 weight for each. Note that Eqs. (7) and (8) are representative variants of additive and multiplicative aggregations for the IPCC’s three components, but some of the variants, not to be covered here, may employ a mixed aggregation scheme of additive and multiplicative forms, such as the MOVE framework.

As summarized in Table 1, the vulnerability to climate change has usually been estimated using one of the six measures from *VA*_1_, *VA*_2_, and *VA*_3_ by additive aggregations, and *VM*_1_, *VM*_2_, and *VM*_3_ by multiplicative aggregations. Therefore, the influence of the six multi-attribute utility forms required for a composite indicator on measuring vulnerability is comprehensively examined as follows.

**Table 1:** Summary of the conventional aggregation frameworks in general forms and equal-weighed forms for vulnerability composite indicators compiled from the IPCC’s assessment components, exposure *E*, sensitivity *S*, and lack of coping *LC* (or coping *C*).

utility function types	aggregation frameworks	
	general forms	equal-weighted forms
additive forms	$V{A}_{1}={\alpha }_{1}E+{\alpha }_{2}S-{\alpha }_{3}C$	$V{A}_{1}=\frac{E+S-C}{3}$
	$V{A}_{2}={\alpha }_{1}E+{\alpha }_{2}S+{\alpha }_{3}LC$	$V{A}_{2}=\frac{E+S+LC}{3}$
	$$\begin{array}{c}V{A}_{3}={\alpha }_{4}PI+{\alpha }_{5}LC,\\ {\rm{w}}{\rm{h}}{\rm{e}}{\rm{r}}{\rm{e}}\,PI={\alpha }_{6}E+{\alpha }_{7}S\end{array}$$	$$\begin{array}{c}V{A}_{3}=\frac{PI+LC}{2},\\ {\rm{where}}\,PI=\frac{E+S}{2}\end{array}$$
multiplicative forms	$V{M}_{1}=\frac{{E}^{{\beta }_{1}}\times {S}^{{\beta }_{2}}}{{C}^{{\beta }_{3}}}$	$V{M}_{1}={(\frac{E\times S}{C})}^{\frac{1}{3}}$
	$V{M}_{2}={E}^{{\beta }_{1}}\times {S}^{{\beta }_{2}}\times L{C}^{{\beta }_{3}}$	$V{M}_{2}=(E\times S\times LC){}^{\frac{1}{3}}$
	$$\begin{array}{c}V{M}_{3}=P{I}^{{\beta }_{4}}\times L{C}^{{\beta }_{5}},\\ {\rm{w}}{\rm{h}}{\rm{e}}{\rm{r}}{\rm{e}}\,PI={E}^{{\beta }_{6}}\times {S}^{{\beta }_{7}}\end{array}$$	$$\begin{array}{c}V{M}_{3}=(PI\times LC){}^{\frac{1}{2}},\\ {\rm{where}}\,PI=(E\times S){}^{\frac{1}{2}}\end{array}$$

### Comparative analysis of vulnerability indicators

To investigate the performance of vulnerability indicators aggregated from the three components of exposure, sensitivity, and lack of coping (or coping) by the six multi-attribute utility functions in Eqs. (3) through (8), after assigning equal weights to all component indicators, the three normalized values of 100.0, 50.5, and 1.0 are chosen for representing high (H), middle (M), and low (L) level scores for each constituent component. Note that 1.0 is taken to represent the low-level score to avoid dividing by zero for coping in Eq. (4). As shown in Table 2, a total of eighteen possible combinations of the three components are implemented for the variation of vulnerability composite indicators. There can exist the six combinations of exposure and sensitivity levels without order of two, H-H, H-M (or M-H), H-L (or L-H), M-M, M-L (or L-M), L-L with respect to the three levels, H, M, and L for lack of coping (or L, M, and H for coping). For example, case (1) comprises three score levels of H-H-H(L) for *E*||*S*, *S*||*E*, and *LC*(*C*) (where “||” denotes “OR”).

**Table 2:** The comparison of vulnerability composite indicators *VA*_1_, *VA*_2_, and *VA*_3_ by additive aggregations in Eqs. (3), (5), and (7), along with *VM*_1_, *VM*_2_, and *VM*_3_ by multiplicative aggregations in Eqs. (4), (6), and (8) with respect to the eighteen reference combination cases of the three score levels high H, middle M, and low L for the three normalized attribute components, exposure *E*, sensitivity *S*, and lack of coping *LC* (or coping *C*).

reference cases	score levels			normalized scores			vulnerability values^a^					
	*E*||*S*	*S*||*E*	*LC*(*C*)	*E*||*S*	*S*||*E*	*LC*(*C*)	*VA*_1_	*VA*_2_	*VA*_3_	*VM*_1_	*VM*_2_	*VM*_3_
(1)	H	H	H(L)	100.0	100.0	100.0(1.0)	100.0	100.0	100.0	100.0	100.0	100.0
(2)	H	M	H(L)	100.0	50.5	100.0(1.0)	75.1	83.5	87.6	79.6	79.6	84.3
(3)	H	H	M(M)	100.0	100.0	50.5(50.5)	75.1	83.5	75.3	27.1	79.6	71.1
(4)	H	M	M(M)	100.0	50.5	50.5(50.5)	50.3	67.0	62.9	21.5	63.4	59.9
(5)	M	M	H(L)	50.5	50.5	100.0(1.0)	50.3	67.0	75.3	63.4	63.4	71.1
(6)	M	M	M(M)	50.5	50.5	50.5(50.5)	25.4	50.5	50.5	17.2	50.5	50.5
(7)	H	H	L(H)	100.0	100.0	1.0(100.0)	50.3	67.0	50.5	21.5	21.5	10.0
(8)	H	L	H(L)	100.0	1.0	100.0(1.0)	50.3	67.0	75.3	21.5	21.5	31.6
(9)	H	L	M(M)	100.0	1.0	50.5(50.5)	25.4	50.5	50.5	5.8	17.2	22.5
(10)	M	L	H(L)	50.5	1.0	100.0(1.0)	25.4	50.5	62.9	17.2	17.2	26.7
(11)	H	M	L(H)	100.0	50.5	1.0(100.0)	25.4	50.5	38.1	17.2	17.2	8.4
(12)	M	M	L(H)	50.5	50.5	1.0(100.0)	0.5	34.0	25.8	13.7	13.7	7.1
(13)	M	L	M(M)	50.5	1.0	50.5(50.5)	0.5	34.0	38.1	4.6	13.7	18.9
(14)	L	L	H(L)	1.0	1.0	100.0(1.0)	0.5	34.0	50.5	4.6	4.6	10.0
(15)	H	L	L(H)	100.0	1.0	1.0(100.0)	0.5	34.0	25.8	4.6	4.6	3.2
(16)	M	L	L(H)	50.5	1.0	1.0(100.0)	−24.4	17.5	13.4	3.7	3.7	2.7
(17)	L	L	M(M)	1.0	1.0	50.5(50.5)	−24.4	17.5	25.8	1.3	3.7	7.1
(18)	L	L	L(H)	1.0	1.0	1.0(100.0)	−49.2	1.0	1.0	1.0	1.0	1.0

^a^normalized values divided by each maximum and multiplying 100.0.

**Figure 1:**
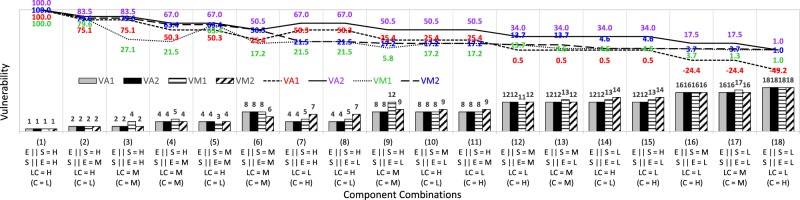
Comparison of variations of vulnerability values and ranking for additive scores *VA*_1_ and multiplicative scores *VM*_1_ aggregated from exposure, sensitivity, and coping components along with those for additive scores *VA*_2_ and multiplicative scores *VM*_2_ aggregated from exposure, sensitivity, and lack of coping components.

**Figure 2:**
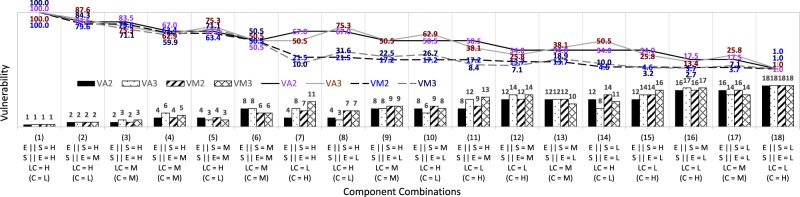
Comparison of variations of vulnerability values and ranking for additive scores *VA*_2_ and multiplicative scores *VM*_2_ aggregated from exposure, sensitivity and lack of coping components along with those for additive scores *VA*_3_ and multiplicative scores *VM*_3_ aggregated from potential impact and lack of coping components.

In Table 2, the eighteen reference cases are arranged from the highest in case (1) to the lowest in case (18) by scoring patterns of the three components. Figures 1 and 2 present the comparisons of vulnerability values (in lines) and ranking orders (in bars) across different aggregation forms, such as the two addictive forms (*VA*_1_ and *VA*_2_) and the two multiplicative forms (*VM*_1_ and *VM*_2_) in Fig. 1, and across different aggregation components such as the three components (VA_2_ and *VM*_2_) and the two components (*VA*_3_ and *VM*_3_) in Fig. 2. Note that in order to approximately present the vulnerability changes between the two discrete reference cases, the vulnerability values for each of the two adjacent cases are linearly connected by lines in Figs. 1 and 2.

Table 2 and Fig. 1 denote the variations of normalized vulnerability indicator, *VA*_1_ and *VA*_2_, by additive aggregations in Eqs. (3) and (5), along with *VM*_1_ and *VM*_2_, by multiplicative aggregations in Eqs. (4) and (6) with respect to the eighteen combinations of the three score levels (H, M, or L) for the three components, exposure *E*, sensitivity *S*, and lack of coping *LC* (or coping *C*). Figure 1 also shows the vulnerability raking orders based on the four kinds of composite indicators, *VA*_1_, *VA*_2_, *VM*_1_, and *VM*_2_, from the three constituent components. As shown in Fig. 1, the ranking from *VA*_1_ and *VA*_2_ values aggregated by the two additive multi-attribute utility functions is completely coincident between the two results. Note that the outcome values of *VA*_1_ and *VA*_2_ can differ (see Table 2 and Fig. 1) in compiling either the coping component for subtrahend or the lack of coping component for adder into the composite indicators by additive multi-attribute utility forms, but the magnitude orders are the same for both indicator outcomes.

Comparing the two multiplicative composite indicators *VM*_1_ and *VM*_2_, some reference cases of the three attribute components show inconsistent outcomes in *VM*_1_, which incorporated the coping component as a divisor. The composite indicator outcomes are expected to be identical for the reference cases that comprise the three components of the same score levels in different orders, such as cases (3) and (2) with H-H-M(M) and H-M-H(L), cases (9) and (10) with H-L-M(M) and M-L-H(L), cases (13) and (12) with M-L-M(M) and M-M-L(H), and cases (17) and (16) with L-L-M(M) and M-L-L(H), respectively. However, the vulnerability outcome of each of the former cases is much lower than that of the latter (i.e. cases (3) < (2), (9) < (10), (13) < (12), and (17) < (16)) as presented in Table 2 and Fig. 1. The vulnerability in reference cases (3), (9), (13), and (17) would be underestimated in *VM*_1_ as a result of a multiplicative aggregation function using a divisor component of coping in Eq. (4). On the other hand, the multiplicative form for *VM*_1_ produces much higher vulnerability in case (5), with M-M-H(L), than case (4), with H-M-M(M) (an equivalent combination to the case (5)) and case (3), with H-H-M(M) (a higher level combination than the case (5)). This is due to the fact that the multiplicative composite indicator *VM*_1_ would generate the overestimated outcomes in such combinations as in case (5), including a low denominator of coping without any comparable low numerators of exposure or sensitivity that cannot cancel out each other. Abnormally large outcomes could lead to ranking reversals in vulnerability assessments measured by *VM*_1_. Such large vulnerability outcomes are also generated in cases (1) and (2), but ranking reversals might not occur since these cases have higher score levels than other reference cases. Note that as shown in Table 2 and Fig. 1, other composite indicators, such as *VA*_1_, *VA*_2_, and *VM*_2_ produce the same vulnerability outcomes between each of the two cases in the above five sets of reference cases, such as cases (2) & (3), (4) & (5), (9) & (10), (12) & (13), and (16) & (17), while there is a difference between each of the two cases in *VM*_1_ outcomes only. Consequently, the vulnerability outcomes *VM*_1_ aggregated from the IPCC’s components *E*, *S*, and *C* by the well-known form of a multi-attribute utility function in Eq. (4) might cause volatility of rankings and rank reversals in decision making.

As denoted in Table 2 and Fig. 1 for comparison of the additive and the multiplicative aggregation functions, the multiplicative composite indicator *VM*_2_ (where excluding *VM*_1_ with some anomalies) generates higher vulnerability scores in case (12) with M-M-L(H) and case (13) with M-L-M(M) than cases (14) with L-L-H(L) and (15) with H-L-L(H). These four reference cases all have the same vulnerability scores in the additive composite indicators *VA*_1_ and *VA*_2_. Case (6) with M-M-M(M) is higher than case (7) with H-H-L(H) in the multiplicative outcomes by *VM*_2_, but case (6) is less than case (7) in the additive outcomes by *VA*_1_ and *VA*_2_. Thus, the outcomes of the additive composite indicators could be compensated with a higher score in any of the three components, while a lower score in any of the three components could drastically reduce the outcomes of multiplicative composite indicators. This is due to the fact that the additive aggregation functions show the higher compensability in that the composite indicators from deficient components can be compensated for by surplus scores of other components, which can hardly compensate for low components in the multiplicative aggregation functions.

Table 2 and Fig. 2 denote the comparison of the vulnerability indicator scores *VA*_2_ and *VM*_2_ aggregated from the three components, exposure, sensitivity, and lack of coping, with *VA*_3_ and *VM*_3_ aggregated from the two components, potential impact (combined from exposure and sensitivity) and lack of coping. The framework comprising the two components with more weighted lack of coping can lead to different vulnerability ranking than that of results combined when the three components are equally weighted. The vulnerability indicators *VA*_3_ and *VM*_3_ can be more sensitive to the lack of coping component weighted at one-half of the exposure and sensitivity components with one-quarter weight for each, given the equal weights of 1/2 as denoted in Table 1. Except cases (1), (6), and (18) with the triplet score levels for the three components, almost all other reference cases show differences in ranking orders between *VA*_2_ and *VA*_3_ by additive aggregations and between *VM*_2_ and *VM*_3_ by multiplicative aggregations. As for the perspective of the lack of coping scores, the vulnerability ranking of *VA*_3_ and *VM*_3_ is higher in cases (5), (8), (10), and (14) with the high level scores of lack of coping, while lower in cases (7), (11), (12), (15), and (16) with the low level scores of lack of coping, compared with that of *VA*_2_ and *VM*_2_, respectively, as shown in Fig. 2.

### Flood vulnerability assessments

Flood vulnerability assessment is one of the essential tools for providing preemptive information on current flood mitigation plans, wherein severe flood damages occur more frequently. Flood vulnerability has been commonly evaluated with a composite indicator compiled from multiple assessment components by easily selecting one of the various aggregation schemes. Although it is natural that different aggregation schemes may generate different composite indicators, it is necessary to understand the impact of different aggregation schemes on vulnerability assessment outcomes. Therefore, a case study on flood vulnerability assessment to climate change is conducted by various aggregation frameworks for the three relevant components in the IPCC TAR^1^.

#### Study site

**Figure 3:**
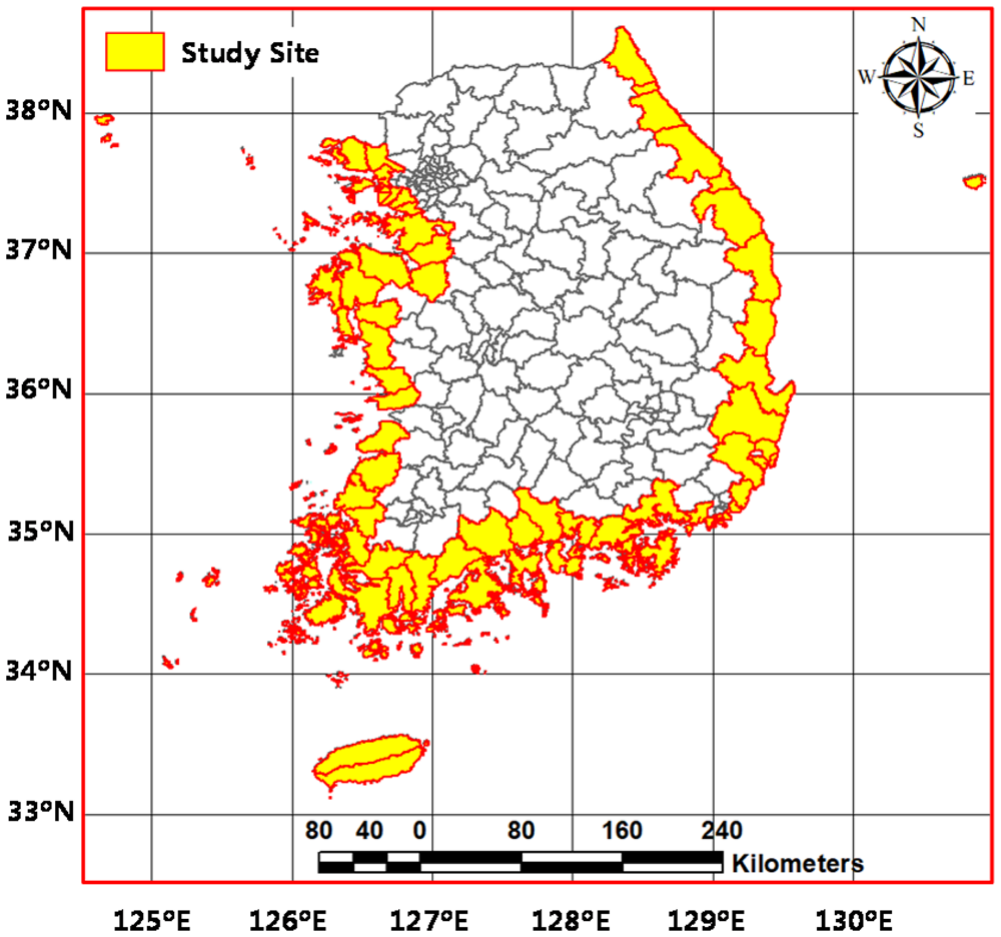
The localization of the coastal 73 administrative districts (yellow) under study in the Republic of Korea.

As shown in Fig. 3, the study site comprises the 73 coastal administrative districts in the Republic of Korea for a comparative analysis of the flood vulnerability ranking orders to climate change across various composite indicators by different aggregation frameworks. The study site is located between 33~43°N and 124~131°E, which extends southwards from continental Asia into the Pacific Ocean. The Korean peninsula is surrounded by the East Sea to the east, the West Sea to the west, and the Korea Strait to the south. The western and southern coasts are irregular with many islands, whereas the eastern coastline is relatively straight. More than half of the annual precipitation falls over the study site between June and September mainly due to the East Asian monsoon and several typhoons. There have recently been severe flood damages in coastal areas of the Republic of Korea due to climate change impacts, as well as geomorphological features.

#### Construction of flood vulnerability indicators

The proxy variables need to be carefully selected and constructed based on the full context of availability, representatives, measurability, integrity, analytical soundness, relevance to phenomena, and relationship to each other^7^. However, there will be some hurdles in the selection of proxy variables for every study on vulnerability assessments despite the best possible efforts and intentions to avoid such issues. In the selection of proxy variables for this study, the main focus was on human and property damage by coastal flooding due to climate change. With reference to previous studies^6,16,17,22,29,33,34^ and data acquisition availability in the study site, this study carefully selected and collected the six proxy variables for the three attribute components of exposure, sensitivity, and coping in the well-known conceptual framework of vulnerability to climate change in the IPCC TAR^1^, as shown in Table 3.

**Table 3:** The selected proxy variables for the coastal flood vulnerability assessment based on the three vulnerability components in the IPCC TAR^1^.

components	symbols	proxy variables	units
exposure	E1	future daily rainfall projections to 2100	mm/day
	E2	100-year storm surge height above ground level	m
sensitivity	S1	children and elderly population density	people/km^2^
	S2	major facilities and infrastructure density	km^2^/km^2^
coping	C1 (or LC1)	emergency facilities density	number/km^2^
	C2 (or LC2)	river improvement ratio	km/km

**Figure 4:**
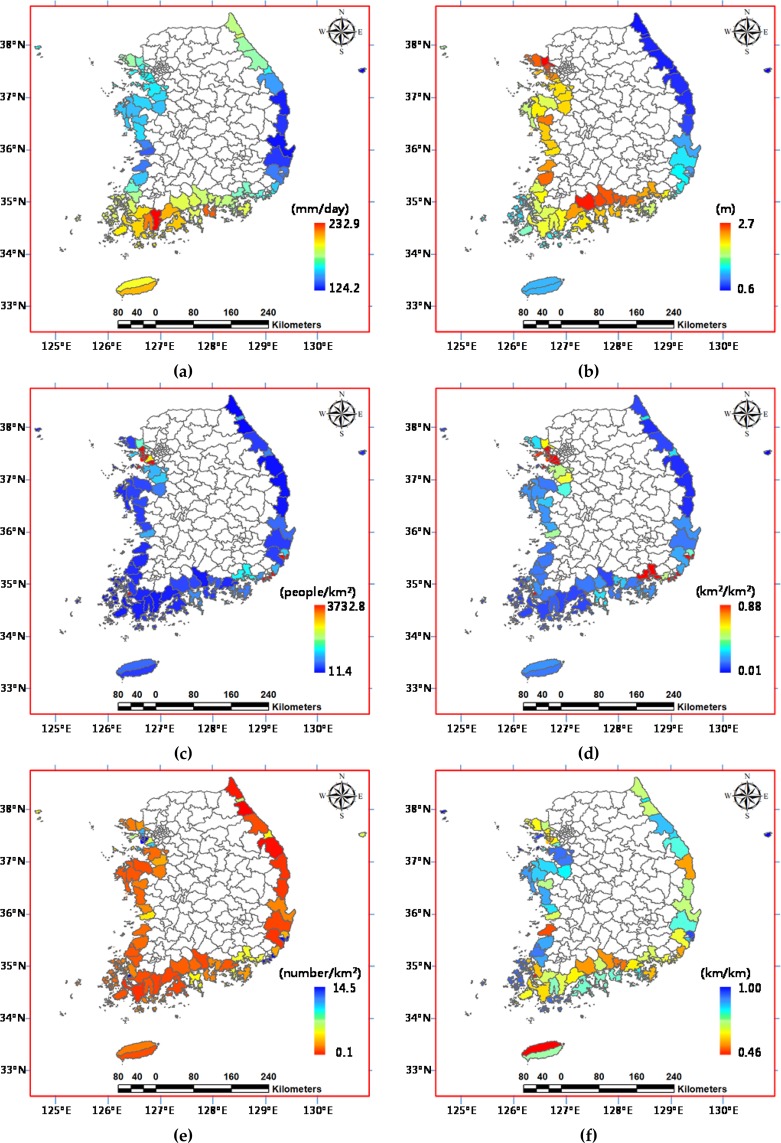
Spatial distribution of the six proxy variables for the flood vulnerability assessment in the Korean Peninsula: (**a**) E1; (**b**) E2; (**c**) S1; (**d**) S2; (**e)** C1 (or LC1); and (**f**) C2 (or LC2).

Due to the lack of data on hydrological modelling for the entire study site, for the geographical range exposed to hazards, the exposure component comprises flood-causing climatic variations in coastal regions, such as the rainfall and storm surge data. Aside from heavy rainfall being the main cause of flooding, storm surge is one of the most dangerous perturbations causing flooding in coastal areas, since it can cause a significant rise in sea level. The future daily rainfall data by climate change based on the IPCC AR5 scenario are collected from the Korea Meteorological Administration Global Atmosphere Watch^35^. The storm surge height data above ground level are constructed from the storm surge data across a 100-year return period using a non-stationary GEV (generalized extreme value) distribution model^36^. The sensitivity component comprises the human and property factors likely to be damaged by hazardous floods, such as the children and elderly population, and major facilities and infrastructure data, which were collected from the Korean Statistical Information Service^37^. On behalf of systems for coping with hazardous floods, the medical and evacuation facilities for emergency services and the river improvement length for flooding countermeasures were selected in the coping component against casualty losses and property damage, respectively. Figure 4 shows the spatial distribution of the six proxy variables in the study site. Overall, the exposure components E1 and E2 are higher in the southern and western parts, with relatively severe climates in low terrains, while sensitivity components S1 and S2 are higher in the north-western and south-eastern parts around metropolitan areas. For the coping components, many districts show the contrary values with lower C1 and higher C2 in general.

For standardization of all the proxy variables in exposure, sensitivity, and coping components, this study uses the min-max normalization method as employed in previous studies^13,14,27,29,38^, which transforms all proxy variables into a common scale between 1 and 100 as follows:

9 $${u}_{i}={s}_{min}+\frac{{x}_{i}-\,\min ({x}_{i})}{\max ({x}_{i})-\,\min ({x}_{i})}({s}_{max}-{s}_{min})$$

where *x*_*i*_ is the original value of the proxy variable *i*, *u*_*i*_ is the standardized value of *x*_*i*_, max(*x*_*i*_) is the maximum value of the proxy variable *i*, min(*x*_*i*_) is the minimum value of the proxy variable *i*, *s*_*max*_ is the standardized maximum value of 1, and *s*_*min*_ is the standardized minimum value of 100.

The lack of coping component for a positive contribution to vulnerability is standardized into reversal values of the coping proxy variables having a negative relationship with vulnerability as used in previous studies^38^:

10 $${u}_{i}={s}_{max}-\frac{{x}_{i}-\,\min ({x}_{i})}{\max ({x}_{i})-\,\min ({x}_{i})}({s}_{max}-{s}_{min})$$

The standardized proxy variables are then compiled into each component indicator *χ*_*j*_ for exposure *E*, sensitivity *S*, coping *C*, or lack of coping *LC* with the weighting factor *w*_*i*_ for the total variable number k as used in previous studies^19,20,27–29,38^:

11 $${\chi }_{j}=(E,S,C,or\,LC)=\mathop{\sum }\limits_{i=1}^{k}{w}_{i}{u}_{i}$$

Finally, the flood vulnerability indicators *VA*_1_, *VM*_1_, *VA*_2_, *VM*_2_, *VA*_3_, and *VM*_3_ are derived by various aggregation frameworks in Eqs. (3)–(8), respectively. Note that the weights are equally assigned to proxy variables *u*_*i*_ and component indicators *χ*_*i*_ in each aggregation scheme in order to prevent unexpected effects of weightings on composite indicator outcomes.

#### Comparison of flood vulnerability indicators

**Figure 5:**
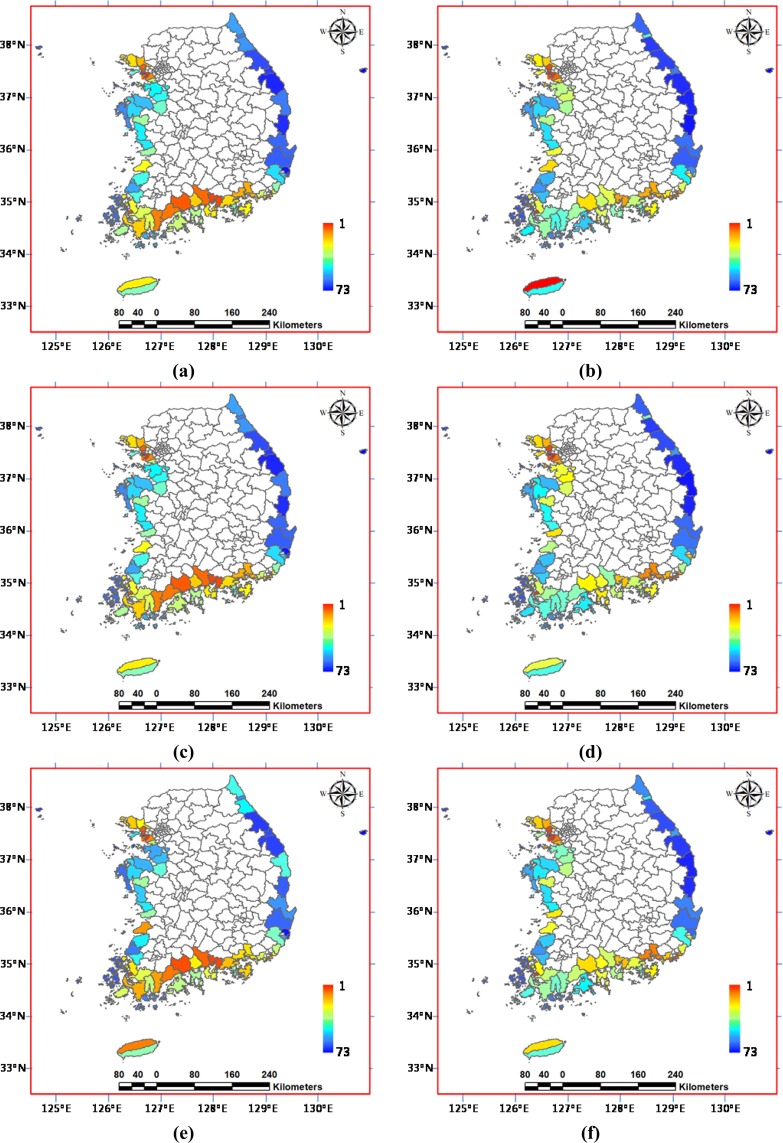
Comparison of the 73 administrative district ranking by the coastal flood vulnerability from various composite indicators: (**a**) *VA*_1_; (**b**) *VM*_1_; (**c**) *VA*_2_; (**d**) *VM*_2_; (**e**) *VA*_3_; and (**f**) *VM*_3_.

The flood vulnerability is ranked for the 73 coastal administrative districts in order of each vulnerability indicator *VA*_1_, *VM*_1_, *VA*_2_, *VM*_2_, *VA*_3_, and *VM*_3_, as shown in Fig. 5. The vulnerability ranking is ordered from higher to lower flood vulnerability outcomes. Figure 5(a,c) denote that regardless of the use of the coping *C* or the lack of coping *LC* component, the flood vulnerability rankings are exactly the same for the study site between the two composite indicator outcomes *VA*_1_ and *VA*_2_ by different additive multi-attribute utility functions in Eqs. (3) and (5). Although the two outcome values of *VA*_1_ and *VA*_2_ can differ in compiling either *C* or *LC* into the composite indicators, no ranking reversal occurs in both outcomes as shown in Table 2 and Fig. 1. In contrast, there are significant ranking changes between the two flood vulnerability outcomes *VM*1 and *VM*_2_ by the two multiplicative multi-attribute utility functions in Eqs. (4) and (6), as shown in Fig. 5(b) and (d). The differences in the vulnerability rankings between *VM*_1_ and *VM*_2_ come from different dispositions in compiling *C* and *LC*, respectively. Figure 5(c,d) denote the comparison between additive and multiplicative composite indicators *VA*_2_ by Eq. (5) and *VM*_2_ by Eq. (6). There are ranking fluctuations between the two flood vulnerability ranking orders in many districts, which are mainly due to the difference of compensability between the two aggregation functions. Figure 5(c,e) denote the comparison of the two additive composite indicators *VA*_2_ from the three components (*E*, *S* and *LC*) by Eq. (5) and *VA*_3_ from the two components (*PI* and *LC*) by Eq. (7). Figure 5(d,f) compare the two multiplicative composite indicators *VM*_2_ from the three components by Eq. (6) and *VM*_3_ from the two components by Eq. (8). Some districts show the apparent changes in the flood vulnerability rankings between *VA*_2_ and *VA*_3_, and between *VM*_2_ and *VM*_3_, respectively, since a more weighted *LC* component can have a larger influence on *VA*_3_ and *VM*_3_.

For further comparative analysis of various flood vulnerability outcomes as shown in Fig. 5, Table 4 also lists the ten selected administrative districts that showed significant ranking fluctuations across various flood vulnerability outcomes, including the normalized values of three assessment components *E*, *S*, and *LC*(*C*), and the flood vulnerability rankings by the six composite indicators *VA*_1_, *VA*_2_, *VA*_3_, *VM*_1_, *VM*_2_, and *VM*_3_. Higher flood vulnerability rankings are determined by the multiplicative aggregation forms *VM*_1_, *VM*_2_, and *VM*_3_, compared to the additive indicators *VA*_1_, *VA*_2_, and *VA*_3_, as shown in districts 2620, 2811, 3114, 4122, and 4127, where the value of neither *E* nor *S* is too low. On the contrary, the flood vulnerability rankings of districts 2611, 4678, 4682, and 4885 are mostly higher in the additive indicators *VA*_1_, *VA*_2_, and *VA*_3_ that have higher compensability by the highest level value for either *E* or *S*, as compared to the multiplicative outcomes by *VM*_1_, *VM*_2_, and *VM*_3_. Table 4 also shows that there is a notable ranking difference between *VM*_1_ and *VM*_2_ in district 2611, which has the three component values comparable to reference case (11) in Table 2. As shown in Table 2 and Fig. 1, the *VM*_2_ produces an identical vulnerability for both reference case (11) with H-M-L(H) and reference case (9) with H-L-M(M), in which both combinations are equivalent to each other. However, the vulnerability of reference case (9) is much lower than that of reference case (11) in *VM*_1_, although the two cases have equivalent scores for the three constituent components. A similar ranking fluctuation between *VM*_1_ and *VM*_2_ occurs in district 5011, ranked as the most vulnerable district by *VM*_1_, where a low denominator value of the coping component *C* will generate abnormally large composite indicator outcomes, as addressed in Section 3. In some districts, the flood vulnerability rankings of the *VA*_2_ and *VM*_2_ aggregated from the three components *E*, *S*, and *LC* with the equal weights of 1/3 are different from those of the *VA*_3_ and *VM*_3_ derived from more weighted *LC* by 1/2 and less weighted *E* and *S* by 1/4. As compared to *VA*_2_ and *VM*_2_, *VA*_3_ and *VM*_3_ showed lower flood vulnerability rankings in districts 2611, 2811, 3144, and 4122, with relatively low values of *LC*, while higher flood vulnerability outcomes were observed in districts 4678, 4682, 4885, and 5011, with relatively high values of *LC*. Such ranking changes appear more prominently in comparing the two multiplicative indicators between *VM*_2_ and *VM*_3_, rather than in the two additive indicators between *VA*_2_ and *VA*_3_. The flood vulnerability outcomes are similar between *VA*_2_ and *VA*_3_ and between *VM*_2_ and *VM*_3_ in districts 2620 and 4127, respectively, with middle level values of *LC*.

**Table 4:** The list of the ten selected administrative districts with significant changes in ranking orders across different flood vulnerability outcomes, along with the three component indicators *E*, *S*, and *LC*(*C*) and the flood vulnerability rankings in the six composite indicators *VA*_1_, *VA*_2_, *VA*_3_, *VM*_1_, *VM*_2_, and *VM*_3_.

district codes	component indicator values			flood vulnerability rankings^a^					
	*E*	*S*	*LC*(*C*)	*VA*_1_	*VA*_2_	*VA*_3_	*VM*_1_	*VM*_2_	*VM*_3_
2611	44.3	100	1.0(100.0)	35	35	44	18	62	72
2620	39.7	54.0	54.2(46.8)	32	32	33	17	14	15
2811	67	18.6	31.0(70.0)	45	45	52	26	21	38
3114	24	42	39.5(61.5)	54	54	58	27	20	32
4122	59.9	13.2	37.9(63.1)	48	48	55	33	26	40
4127	62.7	29.1	53.0(48.0)	36	36	37	19	16	17
4678	95	2.1	77.1(23.9)	10	10	9	39	42	34
4682	81	2.1	80.2(20.8)	20	20	16	40	46	39
4885	87.2	2.1	88.0(13.0)	9	9	8	30	41	33
5011	52.4	4.8	100.0(1.0)	24	24	11	1	30	22

^a^Ranking of 73 coastal administrative districts by flood vulnerability outcomes.

## Discussion

The flood vulnerability assessment is one of the key non-structural measures against flood damage by climate change, a more serious threat in coastal areas than in inland regions due to regional climatic and topographic features. Base on the IPCC’s conceptual framework comprising exposure, sensitivity, and coping components^1^, the vulnerability assessments to climate change have been conducted in various fields, such as human health, ecosystem, water, agriculture, fishery, etc., including floods. This study has therefore assessed the influence of frameworks and utility function forms for constructing a composite indicator on measuring the overall vulnerability to climate change. In the framework for the three vulnerability assessment components in the IPCC TAR^1^, the exposure and sensitivity components have a positive elasticity to the vulnerability, while the vulnerability decreases with the increasing coping score that needs to function as a subtrahend in additive multi-attribute utility forms and a divisor in multiplicative multi-attribute utility forms. Thus, lack of coping with a positive elasticity to the vulnerability like other two components can be used in the opposite concept to coping in the construction framework for composite indicators. The vulnerability ranking orders based on composite indicators *VA*_1_ and *VA*_2_ derived by the two additive aggregation forms are completely coincident regardless of the use of coping or lack of coping component. The two outcome values of *VA*_1_ and *VA*_2_ will naturally differ, but the magnitude sequences are nevertheless unchanged in each vulnerability indicator. However, the volatility of rankings and rank reversals occur in some vulnerability outcomes by a multiplicative multi-attribute utility function with a denominator coping component, since low level coping values will generate abnormally large composite outcomes and middle level coping values will underestimate the vulnerability numerically. Hence, for robustness and confidence in obtaining the vulnerability outcomes by a multiplicative composite indicator, all the assessment components need to have the same directional elasticity to vulnerability. There are also obvious differences in some vulnerability ranking orders between the two composite indicators from additive and multiplicative aggregations. This is due to the fact that additive multi-attribute utility functions will generate composite indicators with higher compensability of attribute components, while multiplicative multi-attribute utility forms will induce one of the low score components to reduce the composite indicator drastically^7^. In the framework for the use of potential impact as a composite component of exposure and sensitivity, the vulnerability indicator may provide misleading information in assigning more weights to the coping component, although these issues can be somewhat mitigated by the use of proper weighting methods as presented in some literature^28,29^.

As shown in a case study involving the Republic of Korea, coastal regions tend to have more severe weather events and more barren areas undeveloped in a natural state or unimproved by forced displacement and planned relocation, as compared to inland regions. On the other hand, most of the densely populated and developed areas along the coastlines have adequately adapted or coped with flooding in the study site. In light of such circumstances, a multiplicative composite indicator *VM*_2_ can be suggested to be more appropriate for measuring the flood vulnerability in the coastal areas under study, given that districts where all of the assessment components are more vulnerable can have higher vulnerability than the ones being compensated for by any larger component. Table 5 reports the top ten administrative districts based on ranking orders by *VM*_2_, as compared to ranking orders by other five flood vulnerability indicators, along with the two selected administrative districts that show extreme fluctuations in ranking orders across different flood vulnerability outcomes in the study site. Although it might be difficult to validate the assessment outcomes of the flood vulnerability to future climate change, the historical flood damage data are collected from the National Disaster Information Center^39^ as a reference for the review on the flood vulnerability results in the study site. Note that for the past five years during 2013 and 2017, the average number of flood damage reports is 6.4 for the top ten administrative districts in Table 5, as compared to 5.2 for the 63 other administrative districts under study.

**Table 5:** The list of the top ten administrative districts based on ranking orders by *VM*_2_ and the two selected administrative districts with ranking fluctuations across different vulnerability indicators, along with ranking orders by other five flood vulnerability outcomes *VA*_1_, *VA*_2_, *VA*_3_, *VM*_1_, and *VM*_3_, accompanied by the three component indicators *E*, *S*, and *LC*(*C*).

district codes	component indicator values			flood vulnerability rankings^a^					
	*E*	*S*	*LC*(*C*)	*VA*_1_	*VA*_2_	*VA*_3_	*VM*_1_	*VM*_2_	*VM*_3_
2817	70.4	88.7	62.9(38.1)	1	1	1	2	1	1
2814	70.6	83.4	60.4(40.6)	2	2	2	5	2	2
2820	70.1	47.6	79.2(21.8)	3	3	3	4	3	3
2650	43.8	91.4	50.8(50.2)	4	4	9	7	4	7
4611	62.9	34.0	87.8(13.2)	6	6	4	3	5	4
2826	77.6	31.1	75.0(26.0)	8	8	7	6	6	5
2629	42.7	63.5	61.5(39.5)	14	14	17	10	7	9
2638	51.1	47.3	65.3(35.7)	18	18	21	11	8	8
2617	45.7	70.2	48.9(52.1)	15	15	26	12	9	14
2818	65.6	28.7	74.9(26.1)	13	13	13	8	10	6
2611^b^	44.3	100	1.0(100.0)	35	35	44	18	62	72
5011^b^	52.4	4.8	100.0(1.0)	24	24	11	1	30	22

^a^Ranking of 73 coastal administrative districts by flood vulnerability outcomes.

^b^Selected districts showing extreme changes in rankings across different flood vulnerability indicators.

Table 5 also demonstrates that the vulnerability ranking results can significantly differ depending on the selection of the vulnerability indicators in this case study. As already mentioned, it is desirable to rank the coastal districts vulnerable to flooding using the multiplicative indicators because higher vulnerability results are expected for the coastal areas where all the assessment components are more vulnerable. However, a multiplicative indicator *VM*_1_ is problematic in that low-level coping values generate undesirable ranking changes as typically denoted in district 5011. The undesirable impact of this problem can also reach the unfavorable ranking alterations in districts 2814, 2650, 2629, 2638, 2617, and 2611. Meanwhile, the additive vulnerability indicators generate higher vulnerability outcomes by one of the much higher-level values in the constituent components, which results in the overestimated districts 2611 and 5011, where one of the assessment components is at the highest level. On the contrary, there are the underestimated vulnerability outcomes by the additive vulnerability indicators in some districts, 2629, 2638, 2617, and 2818, without very high level components. District 2611 has an abnormally higher ranking in *VM*_1_ due to ranking changes with other underestimated districts. It likewise has a higher ranking in *VA*_2_ by compensation of the maximum *S* than in *VM*_2_, which is also higher than in *VM*_3_ with more weights to the minimum *LC*. As low-level coping values will generate undesirable ranking changes in *VM*_1_, district 5011 with the minimum *C* shows the abnormally highest ranking in *VM*_1_. District 5011 also has higher rankings in *VA*_3_ and *VM*_3_, with more weights to the maximum *LC*, compared to rankings in *VA*_2_ and *VM*_2_, respectively. Note that districts 2611 and 5011, with the extreme level coping values, might provide much different flood vulnerability outcomes in the aggregation framework using a denominator coping or a more weighted coping.

This study has compared and evaluated the composite indicators aggregated by the three additive and the three multiplicative utility functions for the IPCC’s vulnerability assessment components. The selection of aggregation frameworks needs to be carefully determined according to the purpose of vulnerability assessments and the characteristics of assessment components, and compensability is one of the basic selection criteria in various aggregation schemes, each with its own benefits and limitations. As an example case, the Unite Nations Development Programme (UNDP) revised the Human Development Index (HDI) by a multiplicative composite scheme, since the additive structure of the original HDI had been criticized for its drawback that allowed for perfect substitution across the three assessment components of health, education, and income^40^. Although both additive and multiplicative aggregation schemes are compensatory schemes, the latter offers lesser compensation for low value components compared with the former with constant trade-offs across all components. In addition, the multiplicative aggregation scheme requires that all components have the same directional elasticity to a composite indicator in order to avoid undesirable ranking reversal problems. As selected for the coastal flood vulnerability to climate change in this study, the multiplicative scheme can provide a better solution to avoid full compensability. Under certain circumstances, however, it would be a partial solution still with some degree of compensability. When it should be interpreted as symmetrical importance of components, it can be solved by a non-compensatory multi-criteria approach^41^. All studies on vulnerability assessments may have limitations and drawbacks in processes for constructing composite indicators. This study employs the min-max normalization method where proxy variables are transformed into a common scale between 1 to 100 and also adopts an equal weighting assumption, the possible weighting systems in lack of a statistical or an empirical basis on causal relationships or consensus of assessment components. Therefore, this comparative case study for various composite indicators needs further investigation on several important processes affecting composite indicator outcomes, particularly for the selection and uncertainty in representative proxy variables for each attribute component, normalization methods for transforming variables measured at different units or scales into a common domain, and weighting schemes for variables and components to contribute to the analysis in different scales.

## Conclusions

As the individual attribute components can be compiled into a composite indicator by multi-attribute utility functions to effectively represent multidimensional complicated issues^8^, the vulnerability to climate change has been generally measured by a composite indicator. A prominent framework for vulnerability assessment to climate change comprises the three attribute components of exposure, sensitivity, and coping, as presented in the IPCC TAR^1^. There has been a variety of aggregation methods for the constituent components into a composite indicator to measure the overall vulnerability to climate change. The conventional frameworks commonly used for aggregating the IPCC’s assessment components can be classified as: 1) an additive form *VA*_1_ and a multiplicative form *VM*_1_ for the three individual components as originally, 2) an additive form *VA*_2_ and a multiplicative form *VM*_2_ for the three individual components by replacing coping with lack of coping, and 3) an additive form *VA*_3_ and a multiplicative form *VM*_3_ for a composite indicator combining the two components, as well as the other one. However, it has not been explicitly interpreted how the construction frameworks and aggregation methods influence the vulnerability composite indicator outcomes. This study has therefore evaluated the robustness and sensitivity of the vulnerability composite indicators aggregated by the conventional additive and multiplicative multi-attribute utility functions from the vulnerability assessment components. The analysis is especially focused on the vulnerability ranking alteration across the six composite indicators by the conventional aggregation frameworks and processes. The effects on the vulnerability outcomes across different construction frameworks are first examined by a design of the possible reference cases of the vulnerability assessment components, and then demonstrated through a case study on the coastal flood vulnerability assessment from actual data to climate change.

It is found that it is desirable for all constituent components to have the same directional elasticity to a composite indicator for robustness of vulnerability scores generated by multiplicative multi-attribute utility forms in order to avoid undesirable ranking reversals. It is also implied that the uncertainty in vulnerability outcomes can increase with the use of potential impact with more weighted coping that tends to have more uncertainty^42^. Multiplicative aggregation allows lesser compensation for low value components whereas additive aggregation provides constant trade-offs among constituent components. When the assessment components are mutually preferentially independent, full substitution should not be allowed across components in general. In such contexts, this study suggests that the coastal flood vulnerability to climate change be evaluated by a multiplicative composite indicator *VM*_2_ from the IPCC’s three components with all the positive elasticity to vulnerability, since regions with a lower component value cannot be fully compensated for by other component values. If trade-offs are not allowed at all, the vulnerability should be assessed by non-compensatory multi-criteria methods. It is important to select a proper framework and aggregation method to construct vulnerability composite indicators, since an undesirable composite indicator outcome due to overlooking irrational features in some aggregations may cause preference reversals in policy and decision making. It should therefore be prudent to select aggregation frameworks for composite indicators with careful consideration of the objectives and features of the vulnerability assessments, as well as the advantages and disadvantages of each aggregation method that may have problematic limitations in construction processes and schemes.
